# Development of Orally Available, Brain Penetrant Compound That Reduces Tau Pathology

**DOI:** 10.1002/alz.095691

**Published:** 2025-01-09

**Authors:** Benjamin Wolozin, Peter E.A. Ash, Amit Berson, Morgan G Stykel, Caroline Murphy, Nick Fredette, Glenn Larsen

**Affiliations:** ^1^ Aquinnah Pharmaceuticals Inc., Cambridge, MA USA; ^2^ Aquinnah Pharmaceuticals, Cambridge, MA USA

## Abstract

**Background:**

Increasing data indicates that the pathophysiology of microtubule associated protein tau is mediated by its interactions with RNA and RNA binding proteins via stress granules (SGs) and the translational stress response. Aquinnah now reports identifying small molecule compounds that inhibit tau/TIA1 SGs in neuronal cell lines and show strong *in vivo* efficacy in a classic mouse model of tauopathy.

**Method:**

Compounds identified using high content imaging screening in SH‐SY5Y neuroblastoma cells, inducibly over‐expressing tau::GFP and TIA1::mKate2, following exposure to stressor. Validation was performed with rat hippocampal primary neurons and 3D human iPSC neuron/astrocyte assembloids. *In vivo* efficacy studies were conducted in 8 & 9‐month old P301S tau mice that were treated daily for 1 month. Pathological tau, misfolded (MC‐1) and phosphorylated (AT8 pS202/T205, pT181), was quantified in treated P301S tau mice by AlphaLISA and immunohistochemistry.

**Result:**

We identified a compound series that reduced tau pathology both *in vitro* and *in vivo*. The lead target acts in the proteostasis pathway, being present in both neurons and immune cells. Strong reductions of tau pathology were observed in primary rat neurons and 3D human iPSC assembloids. Treatment of 9‐month P301S tau mice QD orally for 28 days also showed strong reduction of tau pathology, with results characterized and quantified with AlphaLISAs and immunohistochemistry. Quantification of each marker measured by AlphaLISA showed reductions of: MC‐1, 70% (P = 0.0031), pS202/T205 tau (AT8) 65% (P = 0.0018) and pT181 tau, 60% (P = 0.0005). Histopathology and a clinical chemistry panel indicated no adverse effects from 28‐days of QD treatment.

**Conclusion:**

Aquinnah has identified an orally bioavailable, brain penetrant compound that is able to strongly reduce tau pathology in a mouse model of tauopathy with late stage disease. These results show strong promise as a potential therapeutic agent for treatment of Alzheimer’s disease and related disorders.

